# Supplementary material for: A distinct monocyte transcriptional state links systemic immune dysregulation to pulmonary impairment in long COVID
Source: Nat Immunol. 2026 Jan 14;27(2):200–12. doi: 10.1038/s41590-025-02387-1 (PMC12864029; doi:10.1038/s41590-025-02387-1)
Supplement: Supplementary file 1 — Supplementary Tables 1–5. [file 41590_2025_2387_MOESM1_ESM.pdf]

nature immunology

Supplementary information https://doi.org/10.1038/s41590-025-02387-1

A distinct monocyte transcriptional state links systemic immune dysregulation to pulmonary impairment in long COVID

In the format provided by the authors and unedited

Supplementary Table1: Summary or demographic and clinical measurements for cohort 1, showing mean and standard deviation.

Group	NI	AI-mild/moderate	AI severe	R	LC^{AM}	LC^{AS}
Number of donors (samples)	6	7(7)	4(4)	8(9)	29 (39)	8(13)
Gender/Male	3	4(4)	2(2)	5(6)	12(15)	6(10)
Gender/Female	3	3(3)	2(2)	3(3)	17(24)	2(3)
Age	42 ± 14.45	50 ± 14.17	40 ± 9.66	38 ± 17.96	51 ± 13.16	47 ± 18.40
Partial pressure O2				87.53 ± 8.20	83.04 ± 9.6	84.02 ± 8.41
Partial pressure CO2				39.86 ± 2.69	38.27 ± 2.96	40.04 ± 3.91
FEV1%				96 ± 11.32	98.18 ± 11.44	88 ± 19.95
FVC%				102.55± 11.44	103.05 ± 12.83	84.69 ± 17.63
DLCO%				95 ± 19.81	97.23 ± 17.52	87.46 ± 21.80
TLC%				99.77 ± 6.45	99.20 ± 13.09	84.15 ± 16.14
FAS_Score				15.55 ± 4.66	28.29 ± 11.57	20.84 ± 6.84
MmrcDyspnea_score (samples)				0(9)	0(13),1(17),2(8),3(1)	0(3),1(6),2(3),NA(1)
Quality of Life (QoL,0=bad, 10 = good)					6.23 ± 2.7	7.15 ± 1.7

Supplementary Table2: Summary of demographic and clinical measurements for cohort 2, showing mean and standard deviation.

Group	NI	LC^{AM}	LC^{AS}
Number of donors (samples)	33	117(158)	25(47)
Gender/Male	17	48(65)	20(37)
Gender/Female	16	69(93)	5(10)
Age	41 ± 9.97	49 ± 13.79	48 ± 17.02
Partial pressure O2		84.29 ± 7.81	82.44 ± 8.98
Partial pressure CO2		38.8 ± 2.96	39.92 ± 3.34
FEV1%		98.25 ± 13.19	85.95 ± 21.21
FVC%		103 ± 15.86	86.57 ± 22.62
DLCO%		96.17 ± 16.40	77.82 ± 24.60
TLC%		101.39 ± 13.46	86.10 ± 17.68
FAS_Score		27.02 ± 9.92	22 ± 7.24
MmrcDyspnea_score		0(67), 1(69), 2(20), 3(2)	0(15), 1(22), 2(6), 3(3), 4(1)
Quality of Life (QoL,0=bad, 10 = good)		5.9 ± 2.1	6.7 ± 2.5

Supplementary Table 3 Summary of demographic and clinical measurements for cohort 3, showing mean and standard deviation. All patients were with LC^{AM}

Group	Respiratory PASC	Respiratory PASC with Bronchial Hyper-Responsiveness (BHR)
Acute COVID-severity	Mild (WHO score 1-3)	Mild (WHO score 1-3)
Number of donors	5	3
Gender/Male	2	1
Gender/Female	3	2
Age	41 ± 12.00	50 ±
Partial pressure O2	87.65 ± 11.15	89.62 ± 5.35
Partial pressure CO2	33.53 ± 3.77	37.26 ± 4.5
FEV1%	95.54 ± 5.82	80.71 ± 21.7
FVC%	89.74 ± 9.58	75.61 ± 24.5
DLCO%	85.4 ± 16.57	76.33 ± 10.50
TLC%	106.90 ± 9.89	96.53 ± 7.73
FAS_Score	34 ± 4	32.3 ± 3.5
MmrcDyspnea_score	2(4),3(1)	2(1),3(1),4(1)
ECG	No abnormalities (3)<br>Complete right bundle branch block (2)	No abnormalities (2)<br>incomplete right bundle branch block (1)

Supplementary Table 4 Summary of demographic and clinical measurements for cohort 4, showing mean and standard deviation.

Group	AR-NI	LC^{AM}	LC^{AS}
Number of donors	10	29	11
Gender/Male	4	12	9
Gender/Female	6	17	2
Age	44 ± 12.6	51 ± 11.49	53 ± 15.6
Partial pressure O2		84.91 ± 10.88	75.8± 7.10
Partial pressure CO2		38.84 ± 3.32	41.03 ± 5.9
FEV1%		95.55 ± 15.74	75.45 ± 26.41
FVC%		100 ± 17.74	74.54 ± 24.78
DLCO%		96.65 ± 21.09	69.63 ± 21.18
TLC%		98.34 ± 14.76	78.36 ± 18.46
FAS_Score		30.68 ± 9.87	25.90 ± 10.22
MmrcDyspnea_score		0(8),1(11),2(8),3(2)	0(3),1(3),2(3),3(2)
Quality of Life (QoL,0=bad, 10 = good)		5.2 ± 2.37	5.9 ± 2.7

Supplementary Table 5 Summary of demographic and clinical measurements for cohort 5 (public data) , showing mean and standard deviation.

Group	NI	Non-respiratory PASC	Respiratory PASC
Number of donors	2	4	5
Gender/Male	1	2	3
Gender/Female	1	2	2
Age	77,73	51 ± 11.49	53 ± 15.6
Partial pressure O2		NA	NA
Partial pressure CO2		NA	NA
FEV1%		112 ± 15.89	72.2 ± 12.91
FVC%		107 ± 18.60	67.8 ± 10.25
DLCO%		85 ± 10.95	56 ± 12.66
TLC%		NA	NA
FAS_Score		NA	NA
MmrcDyspnea_score		NA	NA
