# Supplementary material for: A distinct monocyte transcriptional state links systemic immune dysregulation to pulmonary impairment in long COVID
Source: Nat Immunol. 2026 Jan 14;27(2):200–12. doi: 10.1038/s41590-025-02387-1 (PMC12864029; doi:10.1038/s41590-025-02387-1)
Supplement: Supplementary file 2 — Reporting Summary [file 41590_2025_2387_MOESM2_ESM.pdf]

Corresponding author(s): Yang Li

Last updated by author(s): Nov 30, 2025

Reporting Summary

Nature Portfolio wishes to improve the reproducibility of the work that we publish. This form provides structure for consistency and transparency in reporting. For further information on Nature Portfolio policies, see our Editorial Policies and the Editorial Policy Checklist.

Statistics

For all statistical analyses, confirm that the following items are present in the figure legend, table legend, main text, or Methods section.

n/a	Confirmed	
☐	☒	The exact sample size (*n*) for each experimental group/condition, given as a discrete number and unit of measurement
☐	☒	A statement on whether measurements were taken from distinct samples or whether the same sample was measured repeatedly
☐	☒	The statistical test(s) used AND whether they are one- or two-sided<br>*Only common tests should be described solely by name; describe more complex techniques in the Methods section.*
☐	☒	A description of all covariates tested
☐	☒	A description of any assumptions or corrections, such as tests of normality and adjustment for multiple comparisons
☐	☒	A full description of the statistical parameters including central tendency (e.g. means) or other basic estimates (e.g. regression coefficient) AND variation (e.g. standard deviation) or associated estimates of uncertainty (e.g. confidence intervals)
☐	☒	For null hypothesis testing, the test statistic (e.g. F, t, r) with confidence intervals, effect sizes, degrees of freedom and P value noted<br>*Give P values as exact values whenever suitable.*
☒	☐	For Bayesian analysis, information on the choice of priors and Markov chain Monte Carlo settings
☒	☐	For hierarchical and complex designs, identification of the appropriate level for tests and full reporting of outcomes
☐	☒	Estimates of effect sizes (e.g. Cohen's *d*, Pearson's *r*), indicating how they were calculated

Our web collection on statistics for biologists contains articles on many of the points above.

Software and code

Policy information about availability of computer code

Data collection	A detailed description of the data and sample collection can be found in the methods section of the manuscript.<br>EDTA blood was collected from patients which were admitted to the hospital due to COVID-19, as well as from ambulatory patients after SARS-COV-2-infection showing Post-COVID19 symptoms (at least 4 weeks after acute COVID-19) at the Hannover Medical school (MHH) or the Siloah hospital. PBMCs were isolated from whole blood using Ficoll gradient centrifugation. 10x Genomics Chromium Next GEM Single Cell Multiome ATAC + Gene Expression protocol and single cell GEM-X 3` Reagent Kits v4 were used to generate single cell multiome data. Sequencing was performed using the Illumina NovaSeq 6000 platform.<br>The Quanterix HD SP-X Imaging and Analysis System™ was used to measure the plasma samples. Human Corplex cytokine panel 1 10-Plex array was used.
Data analysis	For data analysis, we employed the statistical language R(version > 4).A detailed description of the analysis can be found in the methods section of the manuscript. All code for the analysis is made freely available on github.com/CiiM-Bioinformatics-group/LongCOVID.

For manuscripts utilizing custom algorithms or software that are central to the research but not yet described in published literature, software must be made available to editors and reviewers. We strongly encourage code deposition in a community repository (e.g. GitHub). See the Nature Portfolio guidelines for submitting code & software for further information.

Data

Policy information about availability of data

All manuscripts must include a data availability statement. This statement should provide the following information, where applicable:

- Accession codes, unique identifiers, or web links for publicly available datasets
- A description of any restrictions on data availability
- For clinical datasets or third party data, please ensure that the statement adheres to our policy

Single cell multi-ome data and scRNA-seq data was submitted to EGA and is accessible through following IDs: EGAS50000000142, EGAS50000000143, EGAS0000001215 and EGAS0000001216

Research involving human participants, their data, or biological material

Policy information about studies with human participants or human data. See also policy information about sex, gender (identity/presentation), and sexual orientation and race, ethnicity and racism.

Reporting on sex and gender	Our study included patients of both sexes and therefore applies to both sexes. Sexes were self-reported and later confirmed by DNA genotyping. Single cell dataset from both sexes were equally represented in the datasets.
Reporting on race, ethnicity, or other socially relevant groupings	*Please specify the socially constructed or socially relevant categorization variable(s) used in your manuscript and explain why they were used. Please note that such variables should not be used as proxies for other socially constructed/relevant variables (for example, race or ethnicity should not be used as a proxy for socioeconomic status).* *Provide clear definitions of the relevant terms used, how they were provided (by the participants/respondents, the researchers, or third parties), and the method(s) used to classify people into the different categories (e.g. self-report, census or administrative data, social media data, etc.)* *Please provide details about how you controlled for confounding variables in your analyses.*
Population characteristics	The only relevant population characteristic is the past history of COVID -19 infection. All patients were tested PCR negative at the time of visit and sample collection.
Recruitment	Patients reporting to the pneumological outpatient clinic at Hannover Medical School (MHH, Hannover, Germany) with symptoms such as headaches, dyspnoea and fatigue were recruited based on established LC criteria, 1) Symptoms that persist after acute COVID-19 or its treatment. 2) New symptoms that appear after the end of the acute COVID-19 phase, and can be a consequence of the SARS-CoV-2 infection and 3) Worsening of a pre-existing illness as a result of a SAR-CoV-2 infection. The diagnosis was made in accordance with the German S1 guidelines and the Delphi Consensus Criteria of post COVID-19. The cohort included individuals with all severity of acute COVID-19 (WHO score 1-9).
Ethics oversight	The ethics committee of the Hannover Medical School (MHH) approved the sample collection and analyses (ethics vote 9001_BO_K).Informed consent was obtained from all individual participants included in the study.

Note that full information on the approval of the study protocol must also be provided in the manuscript.

Field-specific reporting

Please select the one below that is the best fit for your research. If you are not sure, read the appropriate sections before making your selection.

☒ Life sciences ☐ Behavioural & social sciences ☐ Ecological, evolutionary & environmental sciences

For a reference copy of the document with all sections, see nature.com/documents/nr-reporting-summary-flat.pdf

Life sciences study design

All studies must disclose on these points even when the disclosure is negative.

Sample size	Samples were chosen to balance the age, gender and acute COVID-19 severity for samples collected for different time points. Where possible, longitudinal samples from same patients were used.
Data exclusions	Single cells with low quality, high mitochondrial gene expression and doublets were excluded from the data analysis of single cell multiome analysis of discovery cohort.
Replication	Findings from single cell data of cohort 1 were vaidated with findings from another single cell study of independent cohort 3 and with publicly available datasets.
Randomization	Samples were randomized to include random 4 different donors for each pool during 10X experiments. Additionally, sample timepoint was randomised along with different donors before 10X experiments.
Blinding	The investigators performed the stratification of the long covid samples to identify differences in long covid disease. Therefore, blinding to group allocations was not possible for the further analysis.

Reporting for specific materials, systems and methods

We require information from authors about some types of materials, experimental systems and methods used in many studies. Here, indicate whether each material, system or method listed is relevant to your study. If you are not sure if a list item applies to your research, read the appropriate section before selecting a response.

Materials & experimental systems

n/a	Involved in the study
☐	☒ Antibodies
☒	☐ Eukaryotic cell lines
☒	☐ Palaeontology and archaeology
☒	☐ Animals and other organisms
☐	☒ Clinical data
☒	☐ Dual use research of concern
☒	☐ Plants

Methods

n/a	Involved in the study
☒	☐ ChIP-seq
☐	☒ Flow cytometry
☒	☐ MRI-based neuroimaging

Antibodies

Antibodies used

For Human:
CD3 SparkBlue SK7 BioLegend 344852
CD14 PacBlue 63D3 BioLegend 367122
CD16 BUV563 3G8 BD 568289
CD51 APC NKI-M9 BioLegend 327913
CD99 PE hec2 BioLegend 398205
CD105 BUV421 43A3 BioLegend 323219
CD120b PE-DAZZLE 3G702 BioLegend 358413
CD163 FITC/PE-CY7 GHI/61 BioLegend 333618/2268070
CD206 APC-CY7/PE-CY7 15-2 BioLegend 321120/321124
CALR purified abcam ab2907
AF488 invitrogen
HLA-DQ BB700 Tu169 BD 745976
HLA-DR AF700 L243 BioLegend 307626
IFNGR1 purified ab154400 ab154400
AF568 invitrogen
IRF8 PE REA516 Miltenyi 130-122-927
TGFB1 PE-CF594 TW4-9E7 BD 562422

Validation

All used antibodies are validated/ quality control tested for the analysis of human cells by flow cytometry according to the manufacturer's information.

Clinical data

Policy information about clinical studies
All manuscripts should comply with the ICMJE guidelines for publication of clinical research and a completed CONSORT checklist must be included with all submissions.

Clinical trial registration

Provide the trial registration number from ClinicalTrials.gov or an equivalent agency.

Study protocol

Note where the full trial protocol can be accessed OR if not available, explain why.

Data collection

Describe the settings and locales of data collection, noting the time periods of recruitment and data collection.

Outcomes

Describe how you pre-defined primary and secondary outcome measures and how you assessed these measures.

Plants

Seed stocks

Report on the source of all seed stocks or other plant material used. If applicable, state the seed stock centre and catalogue number. If plant specimens were collected from the field, describe the collection location, date and sampling procedures.

Novel plant genotypes

Describe the methods by which all novel plant genotypes were produced. This includes those generated by transgenic approaches, gene editing, chemical/radiation-based mutagenesis and hybridization. For transgenic lines, describe the transformation method, the number of independent lines analyzed and the generation upon which experiments were performed. For gene-edited lines, describe the editor used, the endogenous sequence targeted for editing, the targeting guide RNA sequence (if applicable) and how the editor was applied.

Authentication

Describe any authentication procedures for each seed stock used or novel genotype generated. Describe any experiments used to assess the effect of a mutation and, where applicable, how potential secondary effects (e.g. second site T-DNA insertions, mosiacism, off-target gene editing) were examined.

Flow Cytometry

Plots

Confirm that:

☒ The axis labels state the marker and fluorochrome used (e.g. CD4-FITC).

☒ The axis scales are clearly visible. Include numbers along axes only for bottom left plot of group (a 'group' is an analysis of identical markers).

☒ All plots are contour plots with outliers or pseudocolor plots.

☒ A numerical value for number of cells or percentage (with statistics) is provided.

Methodology

Sample preparation

Cryopeserved PBMCs were thawed, stained with the Zombie NIR™ Fixable Viability Kit (Biolegend) at room temperature (RT) in PBS for 15min. Unspecific immunolabeling conferred by Fc receptor binding was blocked by the addition of 10% Gamunex solution (Grifols Deutschland GmbH, Frankfurt am Main, Germany). Surface marker immunolabeling was performed in cell staining buffer (PBS, BSA, EDTA) and Brilliant Stain Buffer (BD), overnight at 4 ◦C with anti-human CD3, CD14, CD16, HLA-DR, CD163 and CD206, CD51, CD99, CD105, CD120b and HLA-DQ antibodies. For intracellular staining, cells were fixed and permeabilized (BD) for 30min at RT, immunolabeling of intracellular markers was performed for 30min in Permwash buffer (BD) at 4°C with anti-human primary CALR, IFNGR1, TGFB1 and IRF8 antibodies. Next cells were immunolabeled with the secondary antibody AF488 and AF568 for 30min in Permwash buffer (BD) at 4°C to label the unconjugated antibodies CALR and IFNGR1, respectively. All donors were also immunolabeled with the correspondent isotype controls for the used antibodies. Cells were washed with PBS.

Instrument

The samples were acquired on a Sony spectral analyzer (ID7000, Sony).

Software

The samples were analyzed with the FlowJo software (10.10.0 Tree Star).

Cell population abundance

Monocytes correspond to roughly 5-10% of the PBMC samples, with a loss of 30% during freeze-thawing process. Purity of monocytes was determined by the expression of CD14+, CD16+ and HLA-DR surface markers.

Gating strategy

SSC-A/FSC-A gate to select monocytic cells -> FSC-H/FSC-A gate to select single cells -> Live/Dead gate to select viable cells -> CD3- gate to exclude T cells -> HLA-DR gate to exclude NK cells -> CD14+/CD16+ gate to select classical (CD14+), non-classical (CD16+), and intermediate (CD14+/CD16+) (see also Supplementary Fig. 5c).

☒ Tick this box to confirm that a figure exemplifying the gating strategy is provided in the Supplementary Information.
